# Spatial-Temporal Variability of Soil Organic Matter in Urban Fringe over 30 Years: A Case Study in Northeast China

**DOI:** 10.3390/ijerph17010292

**Published:** 2019-12-31

**Authors:** Hongbin Liu, Shunting Li, Yuepeng Zhou

**Affiliations:** 1 College of Land and Environment, Shenyang Agricultural University, Shenyang 110866, China; liuhongbinsy@syau.edu.cn (H.L.); 2018220403@stu.syau.edu.cn (S.L.); 2 School of Public Affairs, Xiamen University, Xiamen 361005, China; 3 College of Public Administration, Nanjing Agricultural University, Nanjing 210095, China

**Keywords:** soil organic matter variation, spatial-temporal analysis, farmers’ land use behaviour

## Abstract

The study on soil organic matter (SOM) is of great importance to regional cultivated land use and protection. Based on data collected via continuous and high-density soil samples (0–20 cm) and socio-economic data collected from household survey and local bureau of statistics, this study employs geostatistics and economic statistical methods to investigate the spatial-temporal variation of SOM contents during 1980–2010 in the urban fringe of Sujiatun district in Shenyang City, China. We find that: (1) as to temporal variation, SOM contents in the study sites decreased from 30.88 g/kg in 1980 to 22.63 g/kg in 2000. It further declined to 20.07 g/kg in 2010; (2) in terms of spatial variation, the closer to city center, the more decline of SOM contents. Contrarily, SOM contents could even rise in outer suburb area; and (3) SOM content variation may be closely related to human factors such as farmers’ land use target and behaviour including inputs of chemical and organic fertilizers, types of crops and etc. These findings are conductive to grasp the overall trend of SOM variation and the influence of farmers’ land use behaviour on it. Furthermore, they could provide support for policymakers to agricultural planning and land use monitoring, which consequently aids the improvement of soil quality and food production in the urban fringe areas.

## 1. Introduction

As the world’s most populous country, China has been making great efforts to promote the domestic economic development and ensure global food security. To achieve this goal, the protection of cultivated land plays a crucial role [1,2]. Although China has proposed the strictest cultivated land protection system in the world [3], the actual effect of cultivated land protection is not optimistic. The status of cultivated land quality and ecological protection has become the major constraints of the security of grain and food production [4,5,6]. As one of the important indicators expressing soil quality and fertility, soil organic matter (SOM) is not only the main component of organic and inorganic colloidal complex, but also the source of mineral and organic nutrients for plants [7]. Moreover, it is also one of main causes of global climate change [8,9]. SOM not only can store energy, it is also a mediator in the cycling of natural elements, which promotes the cycling and transformation of soil nitrogen (N), phosphorus (P), sulfur (S), potassium (K) and other nutrients which are of critical importance in environmental protection, soil conservation, sustainable agricultural development, and etc. [10,11,12,13]. The variation of SOM contents could be affected by both natural and human factors [14,15,16]. Therefore, study on SOM has long been a hot issue in the fields of soil science, land science, environmental science and social science [17].

A number of recent studies have investigated the spatial variability of soil and other land properties. Most of them focus on the discussion on soil physical properties [18,19] and the spatial variation of soil salinity [20] from the perspective of natural science. As the research further develops, the geostatistical study of the spatial variability of soil nutrients is increasing [9,21,22], for example, the study of SOM [23,24], total N, NO^−^_3_-N, total P, available P, available K [25,26,27], nugget effect and degree of correlation [28,29,30], variable coefficient, etc. [31,32]. However, the extant study may be improved in the following aspects. First, regarding the study objects, most current study concentrates on one time-point and one particular area. Study on both the spatial and temporal variability of soil nutrition contents, especially based on long-term and continuous monitoring data, is scant. Some exceptions are studies of Li et al. [14], Wang et al. [33] and Guo et al. [34]. Second, regarding the study sites, few studies have explored the variability of soil nutrition contents in the urban fringe area. Due to the locational conditions and urban economic radiation, urban fringe has become a place of strategic importance and a place with most land use changes [8,35,36]. Cultivated land protection is no doubt one of the most sensitive and vibrant issues relating to urban fringe [37,38,39,40]. In these regions, on the one hand, farmers are able to take advantage of the access to city markets and thus tend to cultivate high-value crops (e.g., vegetables and fruits) to increase household income [38,41]. On the other hand, farmers in these regions are facing the challenge of relinquishing their cultivated land to accommodate urban expansion [37]. As such, the study on the cultivated land use and protection in urban fringe is of great significance.

Filling these gaps is of great significance to agricultural sustainability. The objective of this study is to explore how SOM contents have changed in time and space in urban fringe where land use changed most dramatically. Answering this question can uncover the spatial-temporal variability of SOM, which may not only provide technical guidance for regional cultivated land use and protection, but also offer policy reference for the trinity protection of cultivated land in quantity, quality and ecology.

This rest of this paper is organized as follows: Section 2 establishes a conceptual framework linking SOM variation to farmers’ land use behavior. Section 3 introduces the datasets, geostatistical and economic statistical methods. Section 4 presents the results. Section 5 discusses the results and Section 6 draws the conclusion.

## 2. Conceptual Framework

**Figure 1:**
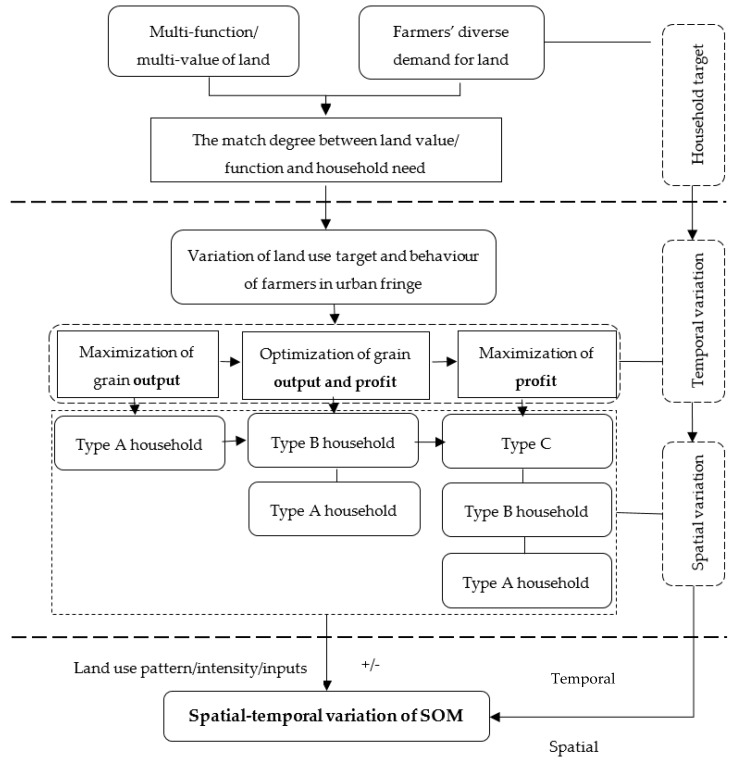
The impact of farmers’ land use behaviour on spatial-temporal variation of SOM in urban fringe.

On the theoretical basis of development economics, peasant household economics, soil science and land resource management, farmers’ understanding of the characteristics of cultivated land is a process with continuous perception and cognition [42]. To reach utility maximization, farm households generate two kinds of demand for cultivated land: demand for grain production (food demand) and demand for economic-value (profit demand) [43]. Households’ demand for land could be affected by the differences in urbanization level, market environment, economic development level and population pressure [44,45,46]. As such, their land use targets could also be diversified [47]. Correspondingly, farmers’ land use target has been shifting from ‘maximization of grain output’, ‘optimization of grain output and profit’ to ‘maximization of profit’ since the reform and opening-up policy in China (see Figure 1). Therefore, farmers’ land use target presents some spatial-temporal variation. Farmers per se evolve from pure farmers (Type A) (Note: Type A household refers to household whose income mainly comes from farming; Type B household refers to household who has concurrent off-farm employment, but farming is still the major income source; and Type C household refers to household who has a concurrent off-farm employment, but off-farm income becomes the major income source) to concurrent farmers who focus on farming (Type B) and then to concurrent farmers who focus on off-farm employment (Type C). On this basis, the spatial-temporal variation of SOM contents is likely to be closely related to the evolvement of the type of farmers. This can be elaborated as follows.

Firstly, when the regional economic level is at a relatively low stage of development (say 1980s), the primary goal of farmers’ land use is to meet the basic needs for survival, that is, to meet the family’s demand for food. The agricultural products (mainly grain crops) produced are mainly consumed by rural households themselves. At this stage, off-farm job opportunities are rare. Farm income is often the most important or even the only source of income for farmers. In order to get higher yield out of land, farmers invest a lot of time and labor on land. To improve soil fertility, manure is widely used. However, due to the limits of land-conservation investment, technical and management levels, and the intensive use of land together made the SOM contents and soil fertility decline.

Secondly, thanks to urban expansion, economic development and the increase of per capital GDP since the beginning of the 21st century, farmers in urban fringe were able to obtain more and more off-farm employment opportunities [48,49]. Besides, the development of urban cities requires large amount of food such as fresh vegetables, which provided a chance for farmers in urban fringe to adjust their planting structure that used to be concentrating on grain production. Under such circumstance, farmers could modify their land use patterns so as to meet the twofold goals, i.e., the pursuit of the maximization of both basic food demand and profit. At this time, the land use behavior of farmers starts to differentiate. The land use pattern of farmers fluctuates between food crops and cash crops.

Compared with the first stage, farmers’ input and management level have been greatly improved, thus the reduction of SOM contents has been inhibited. However, due to the opportunity cost, farmers are not willing to concentrating in farming. As a result, farmers have insufficient incentives to improve the soil fertility and thus SOM contents may also decline. Therefore, during this period, SOM contents were bidirectional, depending on the level of urbanization and farmers’ household income. When the farm income exceeds farmers’ expectations, they will make reasonable investment and management on the land, which would be contributive to the improvement of SOM. Conversely, when farmers consider that farming is not cost-effective, a decline in SOM is likely to occur.

Third, as the further improvement of the economy, farmers’ land use target turns into the maximization of profit. The inputs of land, capital, technology and labor force are expected to be most cost-effective. As a result, more and more cultivated land has been used to plant cash crops. Due to the relatively high opportunity cost of labor, the land use behavior of farmers will be further differentiated. A number of smallholders are able to completely leave their land, with their land handed over to large farmers. Large-scale farming has thus been formed. As a result, land use has become more intensive.

In addition, since the reform and opening up, the main changes of farmers’ demand are shown as follows: from basic food demand to solve the problem of food and clothing → monetary expenditure demand (based on the problem of food and clothing) → profit maximization demand. However, the goal of peasant household land use is consistent with the goal of peasant household economic activities. The phased change of farmers’ land use goals leads to the specific performance of farmers’ land use behaviors at different times, namely, the maximization stage of grain output → the optimization stage of grain output and profit → the maximization stage of profit. The spatial distribution pattern of farmers’ land use group behavior appeared in different spatial scopes at the same time: agriculture-oriented farmers → part-time farmers → part-time farmers and agriculture-supplemented farmers. The change of farmers’ land use in terms of land use mode, degree and input intensity is bound to lead to different evolution rules of cultivated soil organic matter in time and space.

## 3. Materials and Methods

### 3.1. Study Sites

We selected Sujiatun district in Shenyang City, the sole megacity in the three provinces in northeastern China, as the study site. Sujiatun district (123°09′–123°47′ E, 41°27′–41°43′ N) is situated in south Shenyang, which is 15 km from the city center and has a total area of 782 km^2^. It is located in the north temperate zone which is characterized by a sub-humid continental monsoon climate, rainy (dry) season coincided with high (low) temperature, abundant sunshine, and concentrating precipitation. The average annual temperature is 8 °C, the average annual precipitation is 659.6 mm, and the annual sunshine is 2527 h. It is located in the transitional zone of Liaodong Hill and Liaohe Plain with topography sloping from the east to the west. The topography of Sujiatun district is complicated: with mountainous area in the east, hilly area in the middle and plain area in the west. The east hilly area takes up 17% of the total area with an elevation varying between 150–250 m. Most of the area is forestland with the slope varying between 15–25°. The middle mountainous area occupies about 33% of the total area with an elevation lying between 70–100 m. It is the major producing area of upland crops and oil plants. The rest 50% of the total area is the west plain, with an elevation of 30–40 m. The alluvial plain in west area is flat which makes it the major zone of modern agriculture. 

**Figure 2:**
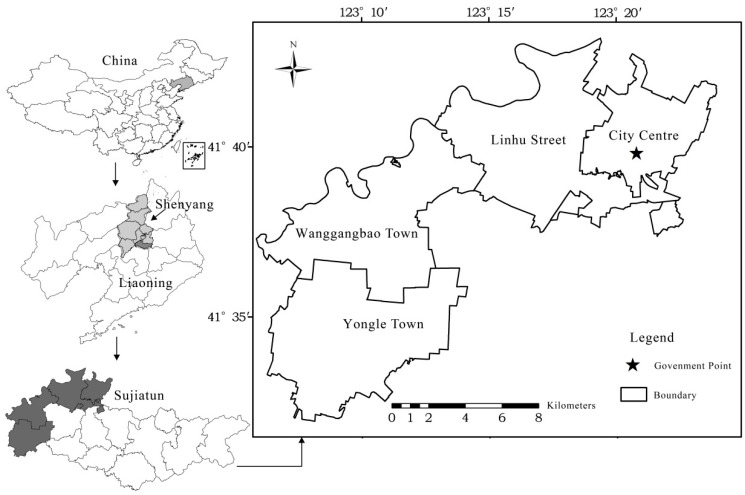
Location of the study area.

Sujiatun district is a demonstration point of the National Commodity Grain Base and Food Self-support Project and Liaoning Standard Agriculture Base. It is one of the main supply locations of vegetables and fruits for Shenyang city. As Figure 2 shows, we selected Linhu street, Yongle town and Wanggangbao town from the west plain area of Sujiatun district. The natural conditions in the three study sites are similar. About 99.99% of the study area is of a slope less than 2 degrees. The major land use type is dry land which accounts for 87.3% of the total cultivated land area in the study sites. The dominant soil in this area is loamy meadow soil, taking up to 89.2% of the total cultivated land area. This guarantees that, under relative homogeneous natural conditions, the variation of SOM contents is driven by human factors. As a result, the selection of the study sites is representative to explore the variability of SOM.

### 3.2. Data Collection and Processing

#### 3.2.1. Soil Sampling and Laboratory Analysis

In order to assure the continuity and comparability of soil sampling, the quantity of sample stationing and point location are selected according to pattern spot of cultivated land, cropping system, crop variety and yield level. Soil data and sample distribution in 1980 are obtained from the Second National Soil Survey which was conducted in May-June with a total of 119 sample points and a scale of soil map at 1:50,000. The soil data in 2000 were collected by our research team in May–June based on the points in 1980 but with an increase of sample size to 141. Similarly, the soil data in 2010 were collected by our research team during May–June based on the points in 2000 with a further expansion of sample size in cultivated-land-concentrating area to 1437. The use of the dataset during multiple periods can also be found in other studies of soil properties [50,51,52]. 

At each sample point, 15–20 surface soil samples at 0–20 cm depth were collected using the chessboard method. The soil samples were well blended, equally divided and put into four 20 × 15 cm plastic bags (1 kg each), which were labeled showing soil sample number, plot name, GPS location, the farmers’ names and addresses, and the date of collection (see details in our other study [53]). Meanwhile, the farm households who owned the selected plots were interviewed to collect their information about land use behaviour, planting pattern, fertilization, irrigation and etc. Compared to extant literature with soil sampling in which only a few soil samples would be collected in one village [54,55,56], we collect continuous and high-density soil samples among the study sites, which could largely increase the accuracy and reliability of the results. 

After removing the plant roots and its residues, stones and dead insect bodies, the soil samples were air-dried in drafty places. The air-dried soil samples were then ground and sieved (0.15–1 mm mesh) to analyze its chemical properties according to the national standard method. SOM contents of the soil samples collected in 1980, 2000, and 2010 were determined according to the dichromate oxidation method [57]. In order to ensure accuracy and reliability, soil sampling and processing methods remain consistent among the three years.

#### 3.2.2. Data Preprocessing

**Figure 3:**
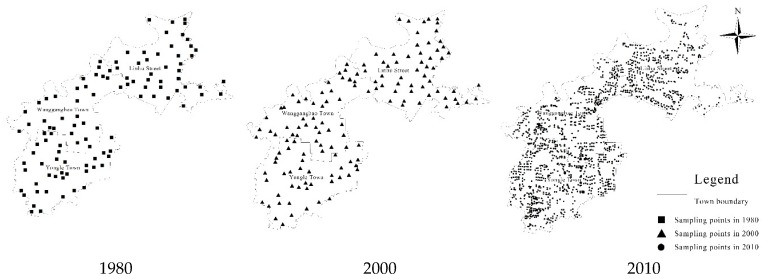
Sample distribution in the study area during 1980, 2000 and 2010.

In China, the land and topographic survey takes Beijing 54 coordinate system as reference, while the GPS data records follow the WGS84 geocentric coordinate system. These two systems have different reference ellipsoids which indicates that the coordinates recorded in the land use maps in 2000 and 2010 need to be transformed. The soil map (1:50,000) in 1980 was drawn by the 3° zone Gauss-Krüger projection system. Thus the GPS data must be projected so as to match the GIS data. In this study, GPS data is read-in by MAPGIS function module. In the process of projection transformation and data format conversion, the GPS data under WGS84 coordinate system is transformed to data under Beijing 54 coordinate system. Then the data with shp format that can be read-in by ArcView is formed. We finally obtain the soil sample distribution maps in 1980, 2000 and 2010, as is shown in Figure 3. The specific steps of this data processing procedure are: 

(1) Employ the soil sampling point data to perform spatial interpolation in GIS. The Kriging interpolation method based on geostatistical theory is used to generate the areal data of the study area. 

(2) Use the GIS partition statistical method to assign the index values of each element of the soil to each plot (note that the plot data is vector space data), and the index used is the soil grid contained in the plot. The attribute data is averaged. Thus, the average value of the soil property data of each plot is obtained. 

For the mathematical statistics, we use SPSS 11.5 (Shenyang Agricultural University, Shenyang, China) to identify outliers. Generally, the threshold value is set by: sample mean ±3 × the mean square error. Then the identified outliers are replaced by the maximum value.

#### 3.2.3. Socio-Economic Dataset

The socio-economic data used in this study mainly come from two sources, one is our first-hand household survey data in 2010, the other is the statistical data provided by the local bureau of statistics. In order to ensure the accuracy of the study and ensure that the data of soil sampling points can correspond to the household survey data one by one, we interviewed farmers while taking soil samples, and recorded their household characteristics, in particular their land use behavior including land use pattern (whether plant cash crops), land use degree (multiple cropping index), and land input intensity (refers to either long-term input such as manure use, or short-term input such as labor/capital input).

To ensure the reliability and representativeness of the household survey data, stratified and random sampling methods were used after the selection of three clusters Linhu street, Yongle town and Wanggangbao town as mentioned above. First, according to the cultivated land area and the typicality of the agricultural production, we use stratified sampling to select villages from the three study sites. Second, we use randomly sampling to select households from the villages. A total of 240 households were interviewed, in which 238 (99.2%) responses were valid. Among them, there are 79 households (33.2%) in Linhu street, 78 households (32.8%) in Wanggangbao town, and 81 households (34%) in Yongle town. The statistical power is 96.2%, which can fully guarantee the reliability of the survey data.

Other socio-economic statistical data mainly come from the local bureau of statistics including: Shenyang Statistical Yearbook (1995–2010), Shenyang Economic Statistical Yearbook (1985–1991), National Economic Data Collection of Sujiatun District (1980–1997), and Statistical Data Collection of Sujiatun District (1998–2010).

### 3.3. Methodology

#### 3.3.1. Geostatistical Method

Semi-variance function, i.e., variogram, is a key function which has been widely used in geostatistics to explore the SOM variability [58,59]. If the random function *Z*(*X*) satisfies the second-order-stationary hypothesis or quasi-intrinsic hypothesis, the spatial structure of each soil property was characterized by experimental semi-variogram *r*(*h*) using the following equation [58]:

(1) $$r\left(h\right)=\frac{1}{2N\left(h\right)}\overset{N\left(h\right)}{\underset{i=1}{\sum }}{\left[Z\left(X_{i}\right)-Z\left(X_{i+1}\right)\right]}^{2}$$

where N(h) represents the number of pairs of observations with an interval of h. 

Parameters such as the nugget C_0_, Sill (C_0_ + C), and their ratio C_0_/Sill are often used in the analysis. The nugget C_0_ is the variation resulted from experimental error and a smaller scale variability of the sampled. The maximum semi-variance found between pairs of points is known as Sill [58]. The smaller the ratio C_0_/Sill is, the larger proportion of structural error (C) is, which implies that soil nutrients were more affected by natural or regional factors. Otherwise, a larger ratio indicates that soil nutrients were more affected by random factors (such as humanity factors). The spatial correlation is strong if the ratio is less than 25%, moderate if the ratio is between 25% and 75%, and weak if the ratio is larger than 75% [60]. 

The Kriging interpolation method is the most widely applied among various interpolation methods in geostatistics. It performs interpolation based on the degree of spatial autocorrelation provided by semivariance analysis [60,61]. In this study, we use GS + win9 (Shenyang Agricultural University, Shenyang, China) and ArcGIS 9.3 (Shenyang Agricultural University, Shenyang, China) to perform the geostatistical analysis. 

#### 3.3.2. Economic Statistical Method

According to the above theoretical analysis framework, we construct the following econometric analysis model:

(2) LUB=f(LUP,LUD,LII)

where *LUB* refers to farmers’ land use behavior, which can be captured by three quantifiable independent variables: land use pattern (*LUP*), land use degree (*LUD*) and land input intensity (*LII*). *LUP* indicates whether farmers grow cash crop, grain crop or both, which states different land use patterns of farmers; *LUD* is represented by the multiple cropping index, which reflects different degree of land use by farmers. The higher the multiple cropping index, the more intensive that land was utilized; *LII* stands for the amount of capital input per unit of land by farmers, which implies the difference of land input intensity among farmers.

The relationship between farmers’ land use behavior and its effects on soil organic matter variation can be represented by:

(3) $$SOM=g\left(LUB\right)=g\left[f\left(LUP,LUD,LII\right)\right]=\alpha +\beta_{1}LUP+\beta_{2}LUD+\beta_{3}LII+\varepsilon$$

where *α*, *β*_1_, *β*_2_ and *β*_3_ are parameters, and *ε* is an error term capturing the unobserved factors.

It should be noted that the above model is a general model of the interaction mechanism between farmers’ land use behavior and the change of soil organic matter. In the specific analysis, according to data availability, we use different data to estimate the model in the temporal and spatial perspective, respectively.

## 4. Results

### 4.1. SOM Variation during 1980–2010

#### 4.1.1. Descriptive Statistics of Soil Organic Matter

The descriptive statistics of the SOM contents in 1980, 2000 and 2010 are reported in Table 1. According to the coefficient of variable (CV), the variability of *SOM* can be categorized as weak (CV < 10%), medium (10% < CV < 100%) and strong (CV > 100%) [17]. The SOM variability in the three periods were all at the medium level. Variability in 1980 was 22.82%, slightly higher than that in the other two periods. As the outliers being replaced, the skewness and kurtosis in all three periods have been decreased. And the K-S tests (>0.05) show that the distribution of SOM in the three periods obey normal distribution.

Over the 30 years, the SOM contents have decreased, though in different declining ranges. According to the grading of SOM contents in the Second National Soil Survey (see Table 2), SOM contents in 1980 (30.88 g/kg) were in grade I, while SOM contents in 2000 (22.63 g/kg) and 2010 (20.07 g/kg) fell in grade III. The annual reduction rate of SOM was 0.41 g/kg during the first 20 years (1980–2000), which was much higher than that during the last 10 years (0.26 g/kg during 2000–2010). 

The maximum and minimum SOM contents in 1980 were 53.87 g/kg and 16.84 g/kg, respectively, with a range of 37.03 g/kg. The maximum and minimum SOM contents in 2000 were 36.19 g/kg and 11.32 g/kg, with a range of 24.87g/kg. And the maximum and minimum SOM contents in 2010 were 27.71g/kg and 17.87 g/kg, with a range of 9.87 g/kg. As can be seen, the range between the maximum and minimum has been declining over the 30 years. The mean differences of SOM contents among the three periods are −8.25, −2.56 and −10.81 g/kg between 1980–2000, 2000–2010 and 1980–2010, respectively, as is shown in Table 3. The *p*-values are all 0.000 which demonstrate that the SOM contents between the three samples are significantly different.

#### 4.1.2. Semivariogram Analysis of SOM Contents

The nugget (C_0_) and Sill (C_0_ + C_1_) in 2000 and 2010 are relatively larger than that in 1980, as is shown in Table 4, indicating that the spatial correlation of the SOM has been declining since 2000. The ratio C_0_/ C_0_ + C_1_ maintains at the range of 25–75% over the 30 years which belongs to the medium spatial variability. This indicates that the spatial variance of SOM contents resulted from both random and structural factors, but the effect of random factors has been strengthening [62]. In particular, the random factors are associated with human activities such as fertilization, cultivation measure, planting system and etc., which could weaken the spatial correlation of SOM. The structural factors are associated with natural conditions such as climate, parent material, topography, soil type and etc., which would strengthen the spatial correlation of SOM. The spatial correlation distances (range) during the three periods are 2560 m, 2040 m and 1480 m, respectively, which indicates a reduction of range over the 30 years. 

#### 4.1.3. Spatial-Temporal Analysis of SOM Variation

**Figure 4:**
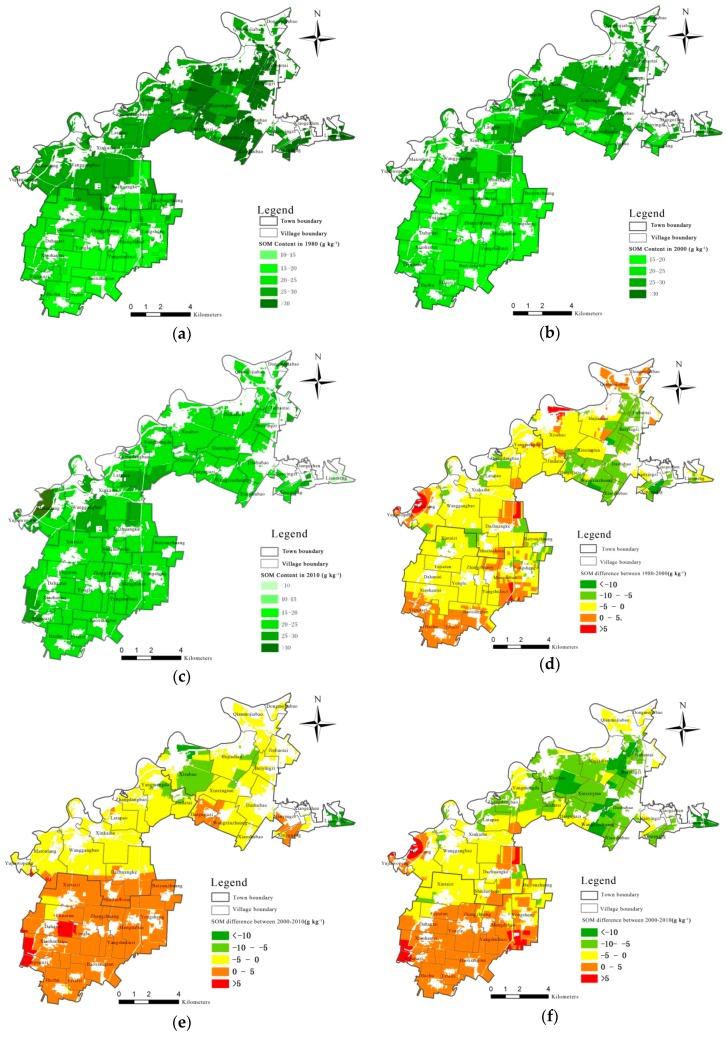
SOM distribution and difference during 1980–2010. (**a**) SOM distribution in 1980 (**b**) SOM distribution in 2000 (**c**) SOM distribution in 2010 (**d**) SOM difference between 1980–2000 (**e**) SOM difference between 2000–2010 (**f**) SOM difference between 1980–2010.

Based on the semivariogram parameters fitted by GS+win9, the grades of SOM contents (Table 5) during the three periods have been interpolated using Kriging method in Figure 4. Regarding the variation of quantity, SOM contents in 1980 had a disperse distribution, with an average of 30.88 g/kg. Most area lied in grade II (25–30 g/kg), which was 4797 hm^2^ and took up 42.8% of the total cultivated land area in Sujiatun district. This area was mainly located in Wanggangbao town and Xisubao village, Xinxingtun village, Qianmojiabao village and Dongmojiabao village in Linhu street. The area with a grade III (20–25 g/kg) SOM ranked second, with an area of 2893 hm^2^ (25.8%), which mainly scattered in the north of Yongle town, including Xintaizi village, Shuiluobo village and Baiyunzhuang village. There were also some areas whose SOM contents fell in grade IV (15–20 g/kg) and I (>30 g/kg), with a proportion of 17.3% and 14.1%, respectively. Jinbaotai village, Beiyingzi village and Dashubao village in Linhu street belonged to this group. As regards to the spatial variation of SOM, as Figure 4a shows, the farther away from the urban area, the lower the SOM contents were.

Figure 4b displays the SOM contents in 2000. The average SOM in 2000 was 22.63 g/kg. The area with SOM in grade I and IV were 121 hm^2^ and 283 hm^2^, respectively, which were 1456 hm^2^ and 1661 hm^2^ less than that in 1980. Most of the area in 2000 concentrated in grade II and III, with an area of 5503 hm^2^ (49.0%) and 5313 hm^2^ (47.3%), respectively. Xisubao village, Hujiadian village, Jinbaotai village, Beiyingzi village in Linhu street and Yangmengda village, Jindatai village and Wanggangbao village in Wanggangbao town were the representatives. The SOM contents in 2010 dropped to 20.07 g/kg. About 84.1% (9433 hm^2^) of the area fell in grade III (see Figure 4c). The SOM contents in 2010 were more homogenous among the region in 2010. 

In general, the SOM contents in the three towns have been declining over the 30 years. The SOM grade has been moving from high and low grades to medium grade. From the perspective of spatial variation, the range of SOM contents variation was −5–0 g/kg during 1980–2000 (see Figure 4d). Villages that presented sharpest decline were Jinbaotai village, Beiyingzi village, Dashubao village, Xiaoshubao village and Xingguang village from Linhu street (suburb of Sujiatun district). However, Huzhu village, Ertaizi village, Yigujiazi village and Baoxiangtun village from Yongle town (outer suburb) had the largest increase of SOM contents by 0–5 g/kg. During 2000–2010, the decline range of SOM in Linhu street and Wanggangbao town was between 0–5 g/kg, whereas the SOM increased by 0–5 g/kg in Yongle town (see Figure 4e). Thus, there was a big difference of the SOM variation between the inner suburb and outer suburb: the SOM decreased by 5–10 g/kg in Linhu street (suburb) and 0–5 g/kg in Wanggangbao town (outer suburb), whereas it increased by 0–5 g/kg in the farthest Yongle town (Figure 4f). Consequently, the closer to city center, the higher range that SOM declined. SOM increased, instead of declining, in the area that is farthest away from city.

From the perspective of spatial difference, the grade of SOM contents in Linhu street in 1980 was highest, mainly falling in grade I and II, with an area of 1552 hm^2^ (13.8%) and 1570 hm^2^ (14.0%), respectively. In 2000, SOM concentrated in grade II (2223 hm^2^, 19.8%). However in 2010, SOM contents declined to grade III (2988 hm^2^). The area with grade I SOM in 2010 sharply declined to 0 and the area with grade II declined by 1465 hm^2^ compared to 1980. In Wanggangbao town, SOM contents in 1980 mainly fixed on grade II (2961 hm^2^, 26.4%). The area of grade II declined by 1337 hm^2^ during 1980–2000 and continued to decrease by 598 hm^2^ during 2000–2010. Although SOM grade in Wanggangbao town was on a declining curve, the range was smaller than that in Linhu street. Another difference is that area with grade I in Wanggangbao town increased by 114 hm^2^ compared to 1980. As regards to Yongle town, SOM contents in 1980 ranged between grade III and IV, with an area of 2296 hm^2^ (20.5%) and 1900 hm^2^ (16.9%), respectively. The area with grade II increased from 2.4% in 1980 to 14.8% in 2000. In 2010, area with grade III was the majority (4030 hm^2^, 35.9%). As can be seen, the trend of SOM variation in Yongle town was different from the other two towns. It demonstrated an upward trend: the area of grade III rose by 1734 hm^2^, while the area of grade IV declined by 1651 hm^2^.

Regarding the spatial variation of SOM, area with SOM declined by 0–5 g/kg were the largest and took up 34.71% of the total cultivated land area. They were mainly distributed in most parts of Wanggangbao town. The second largest area experienced the decline of SOM by 5–10 g/kg (24.47%), which concentrated in most parts of Linhu street. There was still some area witnessing the increase of SOM. Area with SOM rose by 0–5 g/kg ranked top, occupying 31.64% of the total cultivated land area. They were concentrated in most parts of Yongle town. 

As regards to the temporal variation of SOM, as Table 6 shows, during the first 20 years (1980–2000), SOM mostly declined by 0–5 g/kg with a total area of 6744 hm^2^ (60.10%). They were mainly distributed in Wanggangbao town, the south of Linhu street and the north of Yongle town. This was followed by area with SOM declined by 5–10 g/kg (18.16%), which was concentrated in the north of Yongle town and close to fringe of Sujiatun district. Area with SOM increased by 0–5 g/kg took up 2101 hm^2^ (18.72%) and scattered in Huzhu village, Ertaizi village, Yigujiazi village and Baoxiangtun village in the south of Yongle town. During the last 10 years (2000–2010), SOM contents were generally on a declining trend, although in some area they also rose. Area with SOM decreased by 0–5g/kg was 5361 hm^2^ (47.78%), which was mainly located in Wanggangbao town and Linhu street. Area with SOM increased by 0–5 g/kg took up to 42.60% and was mainly concentrated in Yongle town.

To sum up, the spatial-temporal variation of SOM over the 30 years is distinct. From the temporal variation, SOM presents a declining trend and the rate of decline slowed down gradually. As to the spatial variation, the closer to city center, the higher rate of decline of SOM, and the farther away from the city center, the higher rate of increase of SOM. 

### 4.2. The Impact of Land Use Behavior on SOM Variation: A Temporal Perspective

#### 4.2.1. The Change of Farmers’ Land Use Behavior during 1980–2010

Based on the above geostatistical analysis of the SOM variation, in this section, we further examine whether farmers’ land use behavior can explain this variation over the past 30 years in Sujiatun from the aspects of land use pattern (LUP), land use degree (LUD) and land input intensity (LII, manure input). 

(1) Land use pattern (LUP)

**Figure 5:**
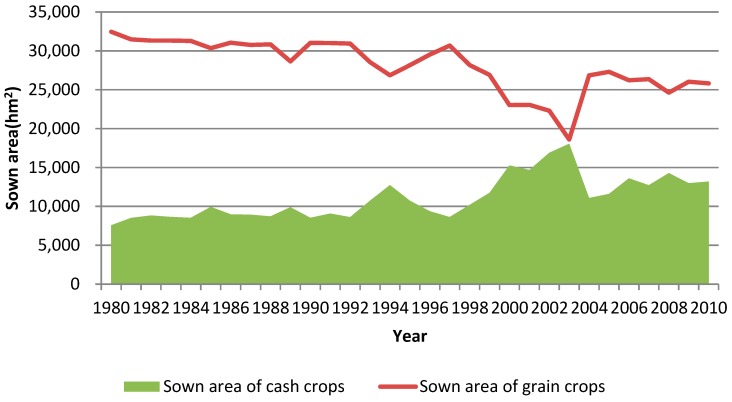
Changing sown areas of grain and cash crops during 1980–2010.

Figure 5 illustrates the sown areas of grain crops (maize and rice) and cash crops (vegetables and fruits), respectively. The sown area of grain crops shows a downward trend as a whole, from 32,457 hm^2^ in 1980 to 25,812 hm^2^ in 2010, declining by 221.5 hm^2^ per year. The decline was relatively obvious since 2000. In 2003, the sown area of grain crops was basically the same as that of cash crops.

**Figure 6:**
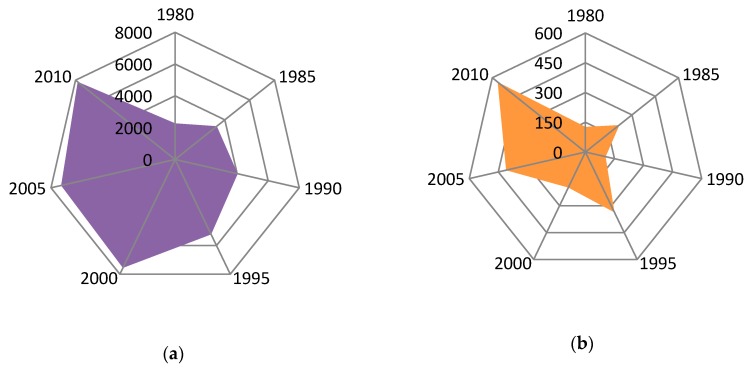
Changing areas of cash crops in Sujiatun during 1980–2010. (**a**) Area of vegitables (**b**) Area of fruits.

Figure 6 shows the change of sown areas for vegetables and fruits, respectively. The change of vegetable planting area has experienced three stages. In the first stage (1980–1990), the area increased slowly from 2271 hm^2^ in 1983 to 3271 hm^2^ in 1990. During this period, the highest value appeared in 1989, which was 6112 hm^2^, and the lowest value appeared in 1984, was 2456 hm^2^. In the second stage (1991–1999), the area increased rapidly from 4030 hm^2^ in 1992 to 8230 hm^2^ in 2000, with an average annual growth of 420 hm^2^. In the third stage (2001–2010), the area fluctuated slightly, first decreased slightly, then increased slowly, and kept at a high level as a whole. During this period, the highest value appeared in 2008 at 8439 hm^2^, and the lowest value appeared in 2004, at 4340 hm^2^.

The change of fruit planting area has also experienced three stages. In the first stage (1980–1990), the area fluctuated at a low level, with an average area of 230 hm^2^. In the second stage (1991–2000), the planting area fluctuated at a medium level, the highest value appeared in 1993, 440 hm^2^, while the lowest value appeared in 1991. In the third stage (2001–2010), the area began to increase rapidly, from 167 hm^2^ in 2001 to 565 hm^2^ in 2010, which increased nearly four times in ten years.

(2) Land use degree (LUD)

**Figure 7:**
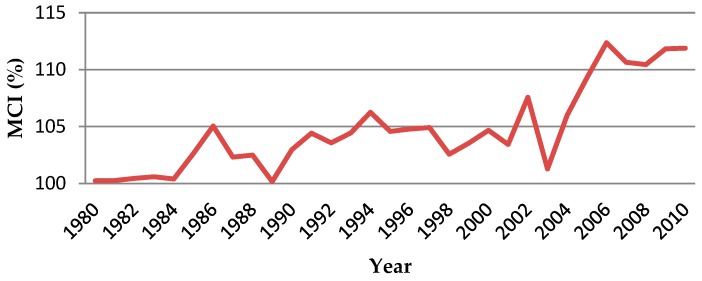
Change of multiple cropping index in Sujiatun during 1980–2010.

As one of the indicators to measure the degree of land use, multiple cropping index (MCI) reflects the impact of population pressure on the planting system under certain natural resources. The statistics of the MCI in Sujiatun district during 1980–2010 (see Figure 7) shows that the degree of land use by farmers has been gradually increasing, which can be roughly divided into three stages. In the first stage (1980–1990), MCI increased from 100.23% to 102.96%. It reached the peak point in 1986, which was 105.04% and touched a trough in 1989 at 100.16%. In the second stage (1991–2000), MCI was basically unchanged, rising from 104.41% in 1991 to 104.67% in 2000. In the third stage (2001–2010), MCI rose sharply from 103.42% in 2000 to 111.88% in 2010. During this period, there was a trough in 2003, which was 101.27%, and a peak in 2006, which was 112.37%. 

(3) Land input intensity (LII, manure input)

**Figure 8:**
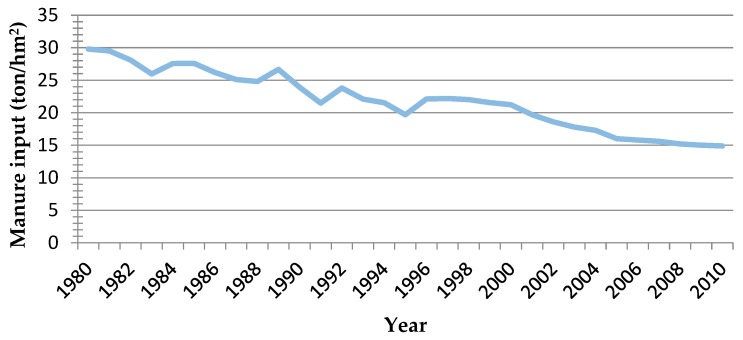
Change of manure input in Sujiatun during 1980–2010.

The input of manure per unit cultivated land shows a downward trend during 1980–2010 as a whole, from 29.8 ton/ hm^2^ in 1980 to 14.9 ton/ hm^2^ in 2010, with a decrease of nearly 50% (see Figure 8). 

However, the trend of the curve shows that from 2004, the decrease of manure input is gradually decreasing. On the one hand, with the rising opportunity cost, it takes time and efforts for the rural labor in the urban fringe to apply manure, so many farmers are reluctant to invest in manure. On the other hand, with the decline of livestock and poultry breeding, many farmers have no manure at all. If they want to use manure, they need to purchase from the market, which inevitably increases the cost of agricultural production. Another finding is that farmers in the study area know that the application of manure would be beneficial for maintaining and improving the soil organic matter contents. However, due to the low comparative benefit of agricultural production, many farmers are not willing to spend their time and efforts on the application of manure, resulting in the decrease of manure input year by year.

#### 4.2.2. Farmers’ Land Use Behavior and the Temporal Variation of SOM during 1980–2010

**Figure 9:**
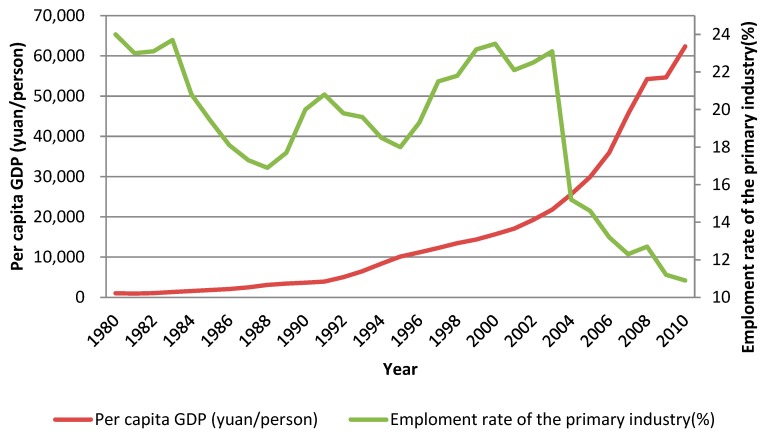
Change of per capita GDP in Sujiatun during 1980–2010.

On the basis of reviewing the change of farmers’ land use behavior, we can now figure out how it might affect SOM’s temporal variation. In the first stage (1980–2000), the contents of SOM in the whole study area tended to decline, from 30.88 g/kg to 22.63 g/kg, with an annual average decrease by 0.41 g/kg. This stage was in the early stage of China’s reform and opening up policy, and the per capita GDP started to rise gradually from a lower level. For example, in 1980, the per capita GDP of the study area was 1013 yuan (as shown in Figure 9). It reached 10,125 yuan in 1995, and 15,666 yuan/person in 2000. However, the ratio of labor force engaged in the primary industry was 24% in 1980 and 23.5% in 2000. Although it fluctuated during this period, the overall situation remained relatively stable. In such an external socio-economic environment, farmers chose to plant maize and rice as their main production activities. The planting area of cash crops was increasing slowly. Thus, the increase of LUD was relatively slow. In terms of land input, although the amount of manure input was at a high level, it was in a downward trend as a whole. Such farmers’ land use behavior directly led to a significant decline in the contents of SOM in the study area. 

In the second stage (2000–2010), the content of SOM in the whole study area continued declining, whereas the decline rate slowed down. By 2010, the content of SOM was 20.07 g/kg, with an average annual decrease by 0.26 g/kg. At this stage, the per capita GDP of the study area increased from 15,666 yuan to 62,357 yuan in 2010, almost quadrupled. And the labor force engaged in the primary industry also began to shift and divide. The proportion of employment in the primary industry decreased from 23.5% in 2000 to 10.9% in 2010. Under the influence of the external environment, the farmers’ land use behavior has also changed greatly. The proportion of cash crops was increasing rapidly. For example, the proportion of cash crops planted in 2003 was basically the same as the area of grain crops. The LUD reached 112.37% in 2006, and the input of manure was also stable at about 15 tons/hm^2^. However, the decrease rate of SOM slowed down, which was mainly due to the continuous planting of cash crops. In order to obtain more benefits, the input of manure in the soil was increased by farmers to improve the soil fertility.

### 4.3. The Impact of Land Use Behavior on SOM Variation: A Spatial Perspective

#### 4.3.1. The Spatial Variation of Farmers’ Land Use Behavior in 2010

(1) Land use pattern (LUP)

Table 7 shows the spatial variation of farmers’ land use behavior in 2010. With regard to crop planting, we find that there were obvious differences in crop selection among farmers from Linhu street, Wanggangbao town and Yongle town. There were 74 households (93.7%) in Linhu street, 23 households (29.5%) in Wanggangbao town and 2 households (2.5%) in Yongle town planting grain crops. The proportion of the total samples planting cash crops were 5.1%, 25.6% and 59.2% among the three sites, respectively. As can be seen, with the increase of distance to city center, the planting area of grain crops was gradually decreasing, while the area of cash crops was the opposite. The planting choices of farmers also differentiated into three types, i.e., grain crop only, cash crop only, and both. 

(2) Land use degree (LUD, multiple cropping index)

As regards the LUD in Linhu street, Wanggangbao town and Yongle town, the spatial difference of MCI also exists. First, among the households with a MCI of 1, 74 households (93.7% of the sample size of Linhu) were from Linhu street, followed by Wanggangbao town (39 households, 50.0%), and Yongle town (2 households, 2.5%). Second, among the households with a MCI between 1.0 and 1.5, Wanggangbao town had the largest number of 26 households (33.3% of the sample size of Wanggangbao), followed by Yongle town (22 households, 27.2%), and Linhu street (3 households, 3.8%). Third, among the farmers with a MCI between 1.5 and 2.0, Yongle town had the highest number of 32 households, accounting for 39.5% of the total sample in this region, followed by Wanggangbao (5 households, 6.4%). No sample households in Linhu street had a MCI between 1.5–2.0 or 2.0–2.5. Fourth, households with a MCI of 2.0–2.5 and 2.5–3.0 were the highest in Yongle town, accounting for 14.8% and 16.0%, respectively. 

(3) Land input intensity (LII, labor and capital inputs)

In our study area, farmers’ land input mainly includes labor and capital inputs. Labor force is divided into family labor and hired labor. Thus this section mainly investigates the difference of farmers’ inputs in family labor, hired labor and agricultural materials such as fertilizers. First, in terms of family labor, most farmers in Linhu street and Wanggangbao town invested 0–10 man-days/mu and 10–20 man-days/mu, respectively. While in Yongle town, the modes of family labor input were 10–20 man-days/mu and over 30 man-days/mu. As can be seen, with the increase of distance from the city center, the amount of family labor input increased gradually. 

Second, with regards to hired labor, its spatial distribution was similar to that of family labor. Around 60% of households in Linhu street and 80% in Wanggangbao town did not hire labor. While nearly 35% of households in Yongle town hired more than 20 man-days/mu. Third, in terms of the capital investment, a majority of the households (91.1%) in Linhu street and nearly half of the households (48.7%) in Wanggangbao town invested only 0–500 yuan of agricultural means of production on per mu of land. However, in the outer suburb Yongle town where cash crops were widely planted, 69% of households invested more than 1500 yuan on per mu of land.

From the above statistical analysis, it can be seen that with the continuous acceleration of industrialization and urbanization, there are obvious differences in land use objectives and types of farmers in the study area. Generally speaking, concurrent farmers who focus on off-farm employment (Type C) are mainly distributed in the inner suburb Linhu street. They usually plant grain crops such as maize which are time- and effort-saving. Their land use degree and land input intensity is relatively low. Pure farmers (Type A) are mainly located in the outer suburb Yongle town. They mainly plant (greenhouse) vegetables, thus they have the highest degree of land use and the largest intensity of land input. Concurrent farmers who focus on farming (Type B) are most located in Wanggangbao town, where lies in the middle of the study area. Farmers here mainly plant corn and vegetables. They present a relatively high degree of land use and great intensity of land input than farmers in the inner suburb. The difference in land use behavior of different types of farmers in different regions will inevitably have different effects on the change of SOM.

#### 4.3.2. The Impact of Farmers’ Land Use Behavior on SOM Variation: An Empirical Estimate

In order to analyze the mechanism of the impact of farmers’ land use behavior on SOM variation in cultivated land, multiple linear regression model is adopted. Using SPSS13.0 software, the analysis results, as shown in Table 8, are obtained. 

From Table 8, it can be seen that different variables representing farmers’ land use behavior had different influence on the change of SOM in different area. Among them, land use pattern (LUP) of farmers had significant and positive impacts on SOM variation in both Linhu street and Wanggangbao town at the 10% significance level. The average contents of SOM would increase by 4.709 g/kg and 4.799 g/kg respectively when farmers from these two areas planted cash crops, ceteris paribus. The impact on SOM variation in Wanggangbao town was slightly greater than that in Linhu street. Land use degree (LUD) had a significant and negative effect on the SOM in Yongle town at the 5% significance level. It means that the SOM contents in this area would decrease by 2.725 g/kg as the increase of LUD by one unit, with other conditions unchanged. However, the SOM content of Yongle town was positively affected by land input intensity (LII). The average increase of SOM content is 0.001 g/kg for each unit increase of land input intensity.

The main reason for the different influence of farmers’ land use behavior on SOM variation in different regions is as follows. Farmers in the inner suburb Linhu street have more non-agricultural employment opportunities. Because of the low comparative benefit of agricultural production, farmers in Linhu mainly grow maize and other grain crops. Therefore, the degree of land use is low, and the intensity of land investment is small. However, farmers in Yongle town, an outer suburb, mainly plant vegetables thanks to the strong demand from urban residents. As a result, land use degree and input intensity in this area are both high. Farmers in Wanggangbao town, however, located in the middle of Linhu and Yongle, which enables farmers in this area choose between grain and cash crops. In Wanggangbao, farmers mainly plant maize and vegetables, with a relatively high land use degree and high land investment intensity. Farmers’ land use behavior forms a circle structure from the city center to the outer suburb, showing a distribution pattern of ‘anti-Thunen circle’.

## 5. Discussion

Compared with previous studies, we employ continuous and high-density soil sampling data to uncover the spatial-temporal variation of SOM in urban fringe over the past 30 years. We also use time series dataset and first-hand survey data to discuss the deep-seated reasons for the variation of SOM from the micro perspective of farmers. In the suburbs of big cities that are most affected by human factors, what kind of changes have taken place in the spatial-temporal variation of SOM and what kind of relationship exists between it and the land use behavior of farmers. 

This study may contribute to the literature both theoretically and practically. First, from the angle of view, this study attempts to establish a bridge between the soil science (natural science) and the economics (social science), and deepen the existing research. Second, from the perspective of research content, this study reveals that the land use behavior of farmers has an impact on the soil from two dimensions, i.e., time and space. The influencing mechanism, the conceptual framework and findings that have been built could provide a good reference for the follow-up research. Third, at the policy level, the urban fringe is the most sensitive area for the development of modern agriculture. Thus, it is helpful for the policy makers to put forward corresponding policies to make clear how farmers’ land use behavior affects SOM content, adjust the economic behavior and agricultural production activities of farmers, so as to promote the sustainable production capacity of soil and environmental sustainability.

Despite of the potential contributions, it should be noted that this study still has the following limitations. First of all, with regard to data collection, this study only obtained the SOM content data of three phases. This made it impossible for us to construct an econometric model to analyze the impact of farmers’ land use behavior on SOM in the time dimension. Moreover, due to the adjustment of the administrative boundaries of the three townships in the Sujiatun, there will be conflict in the statistical caliber. Therefore, this study uses the average of the Sujiatun district as a whole. Secondly, natural factors and human factors are two basic factors driving the change of soil quality of cultivated land. Natural factors affecting the soil quality are mainly topography, climate, parent material and organisms. For example, global warming may have twofold effects on SOM variation. As temperatures rise, on the one hand, soil microorganisms may become more active and could consume non-negligible amount of SOM and thus lead to the depleting SOM. On the other hand, rising temperature is also likely to increase photosynthesis and plant productivity. Litterfall that can be decomposed to SOM is thus expected to increase. As can be assumed, the changes of these factors are the process of accumulation in the medium and long term. They can be regarded as constant compared with the activities of human beings. Therefore, areas that are least affected by natural factors and most affected by human factors are selected as the study sites in this study.

In addition, as the impact of human activities on soil fertility becomes more and more significant, the social, economic, and cultural factors also become important aspects of the soil fertility. As a result, the multidisciplinary and multi-dimensional analysis will be a future direction of soil fertility research. Moreover, as soil fertility is a dynamic and complex change process, how to establish fixed observation points for collecting continuous and accurate data of cultivated land soil fertility and farmers’ land use behavior is a field that needs further research in the future.

## 6. Conclusions

Based on the theories of farmer economics, land resource science and soil science, this study constructs a theoretical framework linking farmer’s land use behavior to the temporal-spatial variation of SOM in urban fringe of Sujiatun district, Shenyang city. The geostatistical and economic statistical results show that: 

(1) From the temporal perspective, during 1980–2010, the total content of SOM in the study area has shown a downward trend, but the rate of decline in different stages is different. From 1980 to 2000, SOM decreased from 30.88 g/kg to 22.63 g/kg, with an annual decrease by 0.41 g/kg. During 2000–2010, it further declined to 20.07 g/kg, with an annual decrease by 0.26 g/kg, which was far less than that in the previous 20 years.

(2) From the spatial perspective, the variability of SOM is significant. The closer to the city center, the greater the decline of SOM was. Conversely, the farther away from the urban area, the smaller the decline was. In some area in the outer suburb, SOM even appeared to rise.

(3) Farmers’ land use behavior, represented by land use pattern, land use degree and land input intensity, show a periodic evolution trend. Specifically, the sown area of grain crops with relatively less intensive land use and profitable has been gradually decreasing. Instead, the sown area of cash crops with relatively more intensive and profitable is gradually increasing. The LUD exhibits a trend of gradual growth, with a significant increase since the beginning of 21th century. Although the application of manure has been declining, the decline trend has been effectively restrained since 2004.

(4) There are obvious differences in farmers’ land use behavior among our study sites. In the inner suburb Linhu street, farmers mainly present a “food demand”, with low land use degree and low land input intensity. In the area between inner and outer suburb, farmers in Wanggangbao mainly present a coordination of “food demand and profit demand”, with a relatively high degree of land use and high intensity of land input. While in the outer suburb Yongle town, farmers mainly show a “profit demand”, which leads to the highest land use degree and land input intensity.

(5) The temporal and spatial variability of SOM is closely related to the change of farmers’ land use behavior. In other words, evolution of farmers’ land use behavior is one of the major driving factors for the change of SOM content in the study area.

## Figures and Tables

**Table 1:** Descriptive statistics of soil organic matter during the three periods.

SOM/g·kg^−1^	Obs.	Mean/g kg^−1^	SD/g·kg^−1^	CV/%	Min/g·kg^−1^	Max/g·kg^−1^	Skewness	Kurtosis	K-S
Measured value in 1980	119	30.88	7.05	22.83	16.84	53.87	1.032	0.934	-
Measured value in 2000	141	22.63	3.53	15.60	11.32	36.19	0.647	−0.139	-
Measured value in 2010	1437	20.07	1.62	14.63	17.87	27.71	0.511	1.187	-
Outliers replaced in 1980	119	30.88	7.04	22.82	16.84	49.76	0.934	0.844	0.69
Outliers replaced in 2000	141	22.63	3.52	15.59	11.32	33.25	0.563	−0.124	0.57
Outliers replaced in 2010	1437	20.07	1.61	14.62	15.67	27.71	0.401	1.067	0.32

**Table 2:** Grades of SOM contents.

Grade	I	II	III	IV	V	VI
SOM/g·k^−1^	>30	25–30	20–25	15–20	10–15	<10

**Table 3:** Differences of the significant test in soil organic matter during three periods.

SOM/g·kg^−1^	Mean	SD	SE	Confidence Interval		*t*	*df*	*p*
				Upper Boundary	Lower Boundary			
SOM1980–SOM2000	−8.25	7.05	0.16	−7.93	−8.57	−50.97	118	0.000 *
SOM2000–SOM2010	−2.56	3.74	0.087	−0.40	−0.73	−6.52	140	0.000 *
SOM1980–SOM2010	−10.81	6.89	0.11	−8.50	−9.13	−55.28	1436	0.000 *

* Note: *p* < 0.005. Data are analyzed using ANOVA method.

**Table 4:** Parameters of best-fitted semivariogram model.

	Model	C_0_	C_0_ + C_1_	C_0_/C_0_ + C_1_	Range/m	R^2^	RSS
SOM1980	Gaussian model	0.02070	0.05780	0.3581	2560	0.798	4.201 × 10^−4^
SOM2000	Gaussian model	0.02351	0.05302	0.4434	2040	0.611	3.835 × 10^−4^
SOM2010	Exponential model	0.0813	0.1636	0.4969	1480	0.732	1.77 × 10^−4^

**Table 5:** Soil organic matter contents level and its distribution.

Region	Grade	SOM (g kg^−1^)	1980		2000		2010	
			Area (hm^2^)	Percent (%)	Area (hm^2^)	Percent (%)	Area (hm^2^)	Percent (%)
Linhu street	I	>30	1552	13.8	121	1.	-	-
	II	25–30	1570	14.0	2223	19.8	105	0.9
	III	20–25	45	0.4	768	6.8	2988	26.6
	IV	15–20	-	-	55	0.5	-	-
	V	10–15	-	-	-	-	1	0.0
	VI	<10	-	-	-	-	74	0.7
Wanggangbao town	I	>30	25	0.2	-	-	139	1.2
	II	25–30	2961	26.4	1624	14.5	1026	9.1
	III	20–25	552	4.9	1730	15.4	2415	21.5
	IV	15–20	44	0.4	228	2.0	1	0.0
Yongle town	II	25–30	266	2.4	1656	14.8	193	1.7
	III	20–25	2296	20.5	2815	25.1	4030	35.9
	IV	15–20	1900	16.9	-	-	249	2.2
	V	10–15	9	0.1	-	-	-	-
Total	I	>30	1577	14.1	121	1.2	139	1.2
	II	25–30	4797	42.8	5503	49.0	1324	11.8
	III	20–25	2893	25.8	5313	47.3	9433	84.1
	IV	15–20	1944	17.3	283	2.5	250	2.2
	V	10–15	9	0.1	-	-	1	0.0
	VI	<10	-	-	-	-	74	0.7

**Table 6:** Areas and percentages of SOM contents variation between different periods.

Period	Item	Variation Range (g/kg)				
		<−10	−10–5	−5–0	0–5	>5
1980–2010	Area (hm^2^)	682	2745	3895	3550	348
	Percent (%)	6.07	24.47	34.71	31.64	3.11
1980–2000	Area (hm^2^)	116	2038	6744	2101	221
	Percent (%)	1.04	18.16	60.10	18.72	1.98
2000–2010	Area (hm^2^)	141	711	5361	4780	227
	Percent (%)	1.26	6.33	47.78	42.60	2.03

**Table 7:** Descriptive statistics of farmers’ land use behavior in the study sites.

Land Use Behavior		Category	Linhu Street		Wanggangbao Town		Yongle Town		Total	
			hh ^1^	%	hh	%	hh	%	hh	%
**Land use pattern (LUP)**		Grain crop only	74	93.7	23	29.5	2	2.5	115	48.3
		Both crops	1	1.3	35	44.9	31	38.3	50	21.0
		Cash crop only	4	5.10	20	25.6	48	59.2	73	30.7
**Land use degree (LUD, indicated by MCI)**		1	74	93.7	39	50.0	2	2.5	115	48.3
		1–1.5	3	3.8	26	33.3	22	27.2	51	21.4
		1.5–2	0	0	5	6.4	32	39.5	37	15.6
		2–2.5	0	0	3	3.9	12	14.8	15	6.3
		2.5–3	2	2.5	5	6.4	13	16.0	20	8.4
**Land input intensity (LII)**	Family labor (man-day/mu ^2^)	0–10	56	70.9	29	37.2	19	23.5	104	43.7
		10–20	19	24.1	40	51.3	26	32.1	85	35.7
		20–30	2	2.5	5	6.4	11	13.6	18	7.6
		>30	2	2.5	4	5.1	25	30.8	31	13.0
	Hired labor(man-day/mu)	0	47	59.9	63	80.8	9	11.1	119	50.0
		1–10	30	38.0	6	7.7	21	25.9	57	23.9
		11–20	2	2.5	5	6.4	23	28.4	30	12.6
		>20	0	0.0	4	5.1	28	34.6	32	13.5
	Capital input per unit of land (yuan/mu)	0–500	72	91.1	38	48.7	9	11.1	119	50.0
		500–1000	6	7.6	17	21.8	7	8.6	30	12.6
		1000–1500	1	1.3	16	20.5	9	11.1	26	10.9
		>1500	0	0	7	9.0	56	69.1	63	26.5

Note: ^1^ hh indicates number of households. ^2^ 1 hectare = 1 hm^2^ = 15 mu.

**Table 8:** Estimate results for the models.

Variable	Linhu Street			Wanggangbao Town			Yongle Town		
	B	T	Beta	B	T	Beta	B	T	Beta
LUP	4.709 *	1.246	0.188	4.799 *	2.603	0.424	--	--	--
LUD	--	--	--	--	--	--	−2.725 **	−2.068	−0.233
LII	--	--	--	--	--	--	0.001 *	1.955	0.215

Note: **, and * indicate significance at the 1%, 5% and 10% level, respectively (-- means insignificant). B value is the coefficient of regression equation. A beta value means the relative weight of each explanatory variable in the model. The greater the absolute value is, the greater the effect of the factor is.
